# Effectiveness of electroacupuncture for polycystic ovary syndrome: study protocol for a randomized controlled trial

**DOI:** 10.1186/s13063-016-1375-4

**Published:** 2016-05-21

**Authors:** Jiao Chen, Shuwei Feng, Jiuzhi Zeng, Xi Wu, Mingxiao Yang, Hongzhi Tang, Huaying Fan, Jie Yang, Fanrong Liang

**Affiliations:** Chengdu University of Traditional Chinese Medicine, Chengdu, Sichuan China; Maternal and Child Health Hospital of Sichuan Province, Chengdu, Sichuan China; Sichuan Orthopedic Hospital, Chengdu, Sichuan China

**Keywords:** Electroacupuncture, Polycystic ovary syndrome, Effectiveness, RCT, Study protocol

## Abstract

**Background:**

Whether electroacupuncture is effective for patients with polycystic ovary syndrome is still inconclusive. Therefore, this study aims to evaluate the add-on effects of electroacupuncture to conventional drugs for the treatment of polycystic ovary syndrome.

**Methods/design:**

This study is a two-center, open-labeled, randomized, controlled trial. A total of 116 eligible patients with polycystic ovary syndrome will be randomly allocated in a 1:1 ratio to the electroacupuncture plus clomiphene citrate group or to the clomiphene citrate group. Participants in the electroacupuncture plus clomiphene citrate group will receive electroacupuncture treatment in addition to clomiphene citrate capsules, whereas participants in the clomiphene citrate group will be prescribed clomiphene citrate capsules only. Electroacupuncture treatment will be performed from the fifth day of menstruation or withdrawal bleeding until the next menstruation, in three sessions per week for three menstrual cycles. The primary outcome is the ovulation rate. The secondary outcomes include the dominant follicle rate, mean number of dominant follicles, endometrial thickness, time point of ovulation, follicular size before ovulation, luteinizing hormone, estradiol level, and pregnancy rate. The measuring points for outcomes will be baseline and the completion of treatment. Any adverse events occurring during the trial process will be recorded. In addition, a quality-monitoring group independent from the research team will be set up to control the quality of the trial.

**Discussion:**

The design and methodological rigor of this trial will allow for the collection of valuable data to evaluate the effectiveness of electroacupuncture for treating polycystic ovary syndrome. Therefore, this trial will contribute reliable evidence for use in clinical decision-making in acupuncture therapy of polycystic ovary syndrome as well as to future research in acupuncture for polycystic ovary syndrome.

**Trial registration:**

Chinese Clinical Trial Registry, ChiCTR-IOR-15007358, registered on 26 October 2015

## Background

Polycystic ovary syndrome (PCOS), resulting from an imbalance of hormones, is a common disease in women. Its clinical manifestations vary, but include reproductive symptoms (hirsutism [1], infertility [2], pregnancy implications, and neonatal complications [3]) and metabolic complications (obesity [4], impaired glucose tolerance, metabolic syndrome [5], and type 2 diabetes mellitus [6]). Usually, many psychological comorbidities accompany PCOS, including depression, anxiety, moodiness, panic attacks, body dissatisfaction, eating disorders, and a lower health-related quality of life [7, 8]. As one of the common causes of anovulatory infertility, PCOS accounts for a significant proportion (40 %) of all women who receive infertility treatment [2]. More than 116 million (3.42 %) women worldwide are affected by PCOS [9]. Using the Rotterdam criteria, the prevalence has been as high as 17.8 ± 2.8 % [10].

Currently, first-line conventional therapy for infertile women with PCOS is the oral administration of antiestrogen agents (for example, clomiphene citrate). Nevertheless, from a clinical point of view, the current management of PCOS with these drugs is not always satisfactory and produces some obvious side effects. After taking clomiphene citrate, metformin, or both, 41 %, 76.3 %, and 31.6 % of women, respectively, still failed to ovulate [11]. The live-birth rate for these patients was only 22.5 % after taking clomiphene citrate, 7.2 % after taking metformin, and 26.8 % after taking both clomiphene citrate and metformin. Among pregnancies, clomiphene citrate caused multiple pregnancies in 6.0 % of the cases. Other side effects include gastrointestinal symptoms, hot flashes, and symptoms associated with ovarian enlargement and ovulation [12].

Whereas first-line conventional therapy is not satisfactory, acupuncture, as a nonpharmacological therapy, is gaining popularity. Findings from previous clinical trials suggest acupuncture may induce ovulation [13]; improve menstrual frequency [14]; and improve depression, anxiety, and health-related quality of life [15] in PCOS patients. Some of the mechanisms underlying acupuncture as a treatment for PCOS have been identified. Acupuncture is able to decrease abnormally high levels of circulating luteinizing hormone (LH), which affect the LH/follicle-stimulating hormone (FSH) ratio [14, 16], and high levels of testosterone [17]. Additionally, the low associated adverse-events rate, a low risk of multiple pregnancies, and the low cost of acupuncture have been noted [18]. However, the methodological quality of these clinical trials varies, resulting in inconclusive statements on the effectiveness of acupuncture for PCOS. In this case, we designed this two-center, open-labeled, randomized controlled trial (RCT) to evaluate the add-on effects of electroacupuncture to conventional drugs on ovulation and some sex hormones in women with PCOS.

## Methods/design

### Overview of the study design

**Fig. 1:**
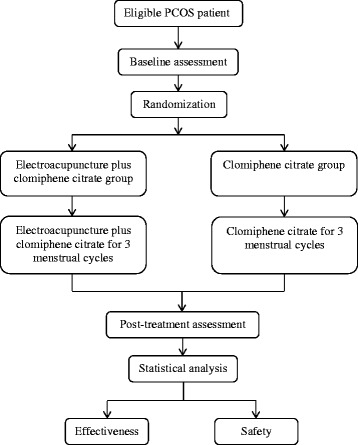
Flow chart of the trial

We designed an open-labeled RCT. The interventions will be performed under ideal circumstances. Therefore, this trial will be carried out in academic settings: the First Affiliated Hospital of Chengdu University of Traditional Chinese Medicine and the Third Affiliated Hospital of Chengdu University of Traditional Chinese Medicine. The flow is shown in Fig. 1.

Before inclusion, participants will be screened in the aforementioned research centers. If the participants meet the inclusion criteria and agree to participate, they will be asked to sign the informed consent form and, then, will be randomized into the electroacupuncture plus clomiphene citrate group (EA group) or the clomiphene citrate group (CC group).

After randomization, the participants will go through a total treatment period of three menstrual cycles. Outcome measurements will be assessed by assessors, blinded to the group assignment, at baseline and the end of treatment. Adverse events will be recorded for the assessment of safety. In addition, a quality monitoring board will be set up to control the quality of the protocol implementation.

The study protocol has been reviewed and approved by local institutional review boards and ethics committees. It follows the principles of the CONSORT and STRICTA statements as well as the Declaration of Helsinki (Sixth revision, 2008). Informed consent will be obtained from all participants.

### Ethics approval

All trial procedures place the participant’s benefit as the highest priority. The present study protocol has already been ethically approved by the Sichuan Regional Ethics Review Committee on Traditional Chinese Medicine with the ethical approval identifier 2015KL-009.

### Participants

We plan to recruit patients with PCOS diagnosed on the basis of *Revised 2003 Consensus on Diagnostic Criteria and Long-Term Health Risks Related to Polycystic Ovary Syndrome* established by The Rotterdam ESHRE/ASRM-Sponsored PCOS Consensus Workshop Group [19]. According to these criteria, women with two out of three of the following items can be diagnosed as having PCOS: (1) oligoovulation or anovulation, (2) clinical and/or biochemical signs of hyperandrogenism, and (3) polycystic ovaries and exclusion of other etiologies (congenital adrenal hyperplasia, androgen-secreting tumors, Cushing’s syndrome). Further assessment of eligibility is based on the inclusion and exclusion criterion as follows.

#### Inclusion criteria

1. Female, age 20 to 40 years
2. Diagnosed as having PCOS
3. Sign the inform consent

#### Exclusion criteria

1. Allergic constitution or allergic to various kinds of medicine
2. Hyperandrogenism due to other reasons, including hyperprolactinemia, thyroid disease, congenital adrenal hyperplasia, Cushing’s syndrome, etc.
3. Pathological endometrial changes, such as uterine malformation, hysteromyoma, etc., diagnosed by B ultrasound
4. Genital tract malformation, gonadal dysgenesis, fallopian tube block, etc.
5. Severe heart, liver, or renal dysfunction or hematological, respiratory, cardiovascular, psychiatric, or other metabolic diseases
6. Within 6 weeks post-abortion
7. Use of hormonal or other medication including Chinese herbal prescriptions in the past 3 months
8. Participation in other clinical trials at the same time

### Randomization

In this trial, participants will be randomly assigned to either the EA group or the CC group in a 1:1 ratio using a table of random numbers. The random numbers are generated using SAS statistical software package (Version 9.1, SAS Institute Inc.).

### Blinding

This is an open-labeled trial; therefore, neither the patients nor the clinical practitioners will be blinded for the entire process. However, in order to eliminate potential bias, other researchers, including data collectors and statisticians, will be blinded.

### Interventions

Participants in the EA group will receive electroacupuncture treatment in addition to the oral administration of clomiphene citrate, whereas participants in the CC group will receive clomiphene citrate only. Treatment in both group will last three menstrual cycles. The intervention in this trial involves a rigorous acupuncture schedule. To ensure the compliance of acupuncturists to the schedule, we ask them to take pretrial training and an entrance exam for this trial. All included acupuncturists will have at least 2 years of practical experience in clinical acupuncture.

#### Clomiphene citrate therapy

On the fifth day of menstruation, all participants, except for those with amenorrhea, will receive a pharmacological treatment regimen of 50 mg clomiphene citrate, taken once each day for 5 continuous days. Patients with amenorrhea will receive the same pharmacological treatment regimen after withdrawal bleeding has been induced by taking progestin. In cases of no ovulation, an additional 50 mg will be added to the dose in the next menstrual cycle. The maximum dose will not exceed 150 mg. The therapy will last for three menstrual cycles.

#### Electroacupuncture

In order to increase the reproducibility and internal validity of the trial, electroacupuncture treatment will be applied to PCOS patients. As the stimulation of electroacupuncture can be more quantifiable relative to manual acupuncture, its employment may reduce the risk of bias somewhat. Sanyinjiao (SP 6), Guanyuan (BL 26) Zhongji (CV 3), and Zigong (EX-CA 1) have been chosen for the formula of compulsory acupoints. Additionally, sets of three arbitrary points will be alternately selected according to the phase of the menstrual cycle: Taichong (LR 3) and Taixi (KI 3) will be needled after menstruation; Mingmen (GV 4) and Xuehai (SP 10), during the ovulatory period; and Xuehai (SP 10) and Geshu (BL 17), before the next menstruation.

Each acupoint will be needled using a filiform needle (25–50 mm in length and 0.25 mm in diameter) to achieve a *Deqi* sensation (refers to a sensation of numbness, distension, or electrical tingling at the needling site and might radiate along the corresponding meridian). Then, an auxiliary needle (13 mm in length and 0.18 mm in diameter) will be inserted, 2 mm lateral to the first needle, to a depth of 2 mm without achieving *Deqi*. Electrical stimulation will be applied at every acupoint, with one electrode connected to the filiform needle and the other to the auxiliary needle. This method circumscribes the electrical stimulation nearby, rather than going across the human body surface to cause performance bias. Electrical stimulation will last for 30 min in each acupuncture session. Electroacupuncture treatment will be performed three times per week from the fifth day of menstruation or withdrawal bleeding until before the next menstruation for three menstrual cycles.

### Outcome measurements

#### Primary outcome

The primary outcome will be the ovulation rate.

#### Secondary outcomes

The secondary outcomes will include the dominant follicle rate, mean number of dominant follicles, endometrial thickness, time point of ovulation, follicular size before ovulation, luteinizing hormone (LH) level, estradiol (E2) level, and pregnancy rate.

#### Time points of outcome measurement

Outcomes will be assessed at two time points: baseline and the completion of the treatment. Furthermore, patients will be given some regular tests at baseline, including routine blood tests, liver function test, and kidney function test. The overview of the outcome measurement at the different time points is shown in Table 1.

**Table 1:** Trial process chart

Time point	Baseline	Post-treatment
Diagnosis		
Inclusion confirmed	✓	
Informed consent signed	✓	
Body sign	✓	✓
Disease history	✓	
Treatment history	✓	
Comorbidity	✓	✓
Current treatment	✓	✓
Outcome assessment		
B ultrasound	✓	✓
Follicle-stimulating hormone	✓	✓
Luteinizing hormone	✓	✓
Testosterone	✓	✓
Estradiol	✓	✓
Blood HCG	✓	✓
Regular tests		
Routine blood tests	✓	
Liver function test	✓	
Kidney function test	✓	
Data collection and statistical analysis		
Adverse event		✓
Causes of dropout		✓
Safety analysis		✓
Compliance analysis		✓

### Assessment of adverse events

According to previous RCTs, acupuncture may cause several types of adverse events, including subcutaneous hematoma, bleeding, skin bruising, and needle site pain. Among these adverse events, subcutaneous hematoma and bleeding were the most common [20]. Acupuncture-related adverse events, including bleeding, hematoma, fainting, serious pain, local infection, etc., will be carefully recorded in the case report forms.

### Sample size calculation

A previous study indicated that 59 % of women with PCOS ovulated after receiving clomiphene citrate [11]. In this trial, the ovulation rate is expected to be 75 % in the EA group and 55 % in the CC group. Considering a significant level of 0.05 and a power of 0.80, 50 participants in each group are required, as calculated by *t* test in G*Power (Version 3, Institute for Experimental Psychology, Heinrich-Heine-University, Germany). At least 58 participants per group and 116 total participants will be recruited to allow for a 15 % dropout rate.

### Statistical analysis

A statistician blinded to the whole trial process will perform the statistical analyses using the SAS statistical software package (Version 9.1, SAS Institute Inc.) in the Computer Integrated Manufacturing System. For the evaluation of a curative effect in this trial, the full analysis set (FAS) will be used. The FAS is determined according to an intention-to-treat (ITT) population; all randomized patients who receive at least one treatment session will be included in the analysis set. The per-protocol analysis set (PPS) is defined as the patients who complete the study and do not have major protocol violations. Demographic data and other basic indicators will be analyzed to test the balance of the two groups at baseline. The main indicators and global indicators will be analyzed within the FAS and PPS. The results will be described with mean, standard deviation, median, P25 (percentile 25), P75 (percentile 75), maximum, and minimum values of the differences between the treatment period and the baseline period. Between-group differences will be tested using repeated measure analyses of the variance. The accepted level of significance for all analyses was *P* < 0.05. Considering the influences of other factors on the effectiveness, such as age, duration of disease, etc., these factors will be considered as covariants for the covariance analysis or logistic regression analysis when comparisons are made between the groups. Missing data will be replaced according to the principle of multiple imputations.

### Quality monitoring

To control the trial’s quality, an independent quality-monitoring group will be established. The project leader will be in charge of the whole quality monitoring, whereas supervisors will be in charge of the quality monitoring in each center. The whole process will be supervised. All data will be verified to ensure they are recorded and reported accurately, authentically, completely, and consistently with the original data.

## Discussion

The present trial is a comparative study of the effectiveness of electroacupuncture on ovulation and some related sex hormones in patients with PCOS.

Due to the weakness of the methodological design, the overall quality of clinical trials concerning the effectiveness of acupuncture treatment for infertility in women with PCOS is relatively low [18]. Although an ongoing multicenter RCT is underway, more RCTs that are properly designed are needed before drawing conclusions concerning the use of acupuncture in the management of PCOS [21]. Therefore, this trial is designed as an RCT to evaluate the add-on effects of electroacupuncture to conventional drugs on PCOS in terms of promoting ovulation and regulating related hormones. This study has several strengths.

On one hand, this trial is designed as an RCT. Due to its ability to reduce spurious causality and bias, the RCT is the most reliable scientific evidence in the hierarchy of evidence and can influence healthcare policy and practice. The RCT, the gold standard in clinical trials, is often used to test the efficacy or effectiveness of various types of medical interventions and may provide information about adverse effects. Therefore, this rigorously designed RCT, which will evaluate the effectiveness of electroacupuncture on PCOS in terms of promoting ovulation and regulating related hormones, will be able to provide some reliable evidence regarding the use of acupuncture for PCOS.

On the other hand, the therapeutic principle of treatment based on the phase of the menstrual cycle will be followed in this trial. In traditional Chinese Medicine, treatment based on syndrome differentiation is an important therapeutic principle. In gynecological diseases, treatment based on the phase of the menstrual cycle is another important principle and can enhance therapeutic effects. Therefore, this principle will be followed in our study. Sanyinjiao (SP 6), Zigong (EX-CA 1), Guanyuan (BL 26), and Zhongji (CV 3) have been chosen as the compulsory acupoints. These four acupoints are the ones most frequently used to treat PCOS, according to a text-mining analysis [22]. Sanyinjiao (SP 6) and Zigong (EX-CA 1) are important acupoints for the treatment of gynecological diseases in acupuncture practice. Guanyuan (BL 26) can reinforce kidney and warm Yang. Zhongji (CV 3) is located in the Conception Vessel and is used to regulate the Chong Vessel and Conception Vessel. In addition, according to a previous study [23], three sets of arbitrary points will be alternately selected according to the phase of the menstrual cycle, with each group consisting of two acupoints. After menstruation, Taichong (LR 3) and Taixi (KI 3) will be punctured to regulate qi and nourish the liver and kidneys. During the ovulatory period, Mingmen (GV 4) and Xuehai (SP 10) will be punctured to tonify qi and regulate the blood to promote ovulation. Before the next menstruation, Xuehai (SP 10) and Geshu (BL 17) will be punctured to regulate the blood.

This rigorously designed trial will allow for the collection of valuable and reliable data to evaluate the effectiveness of a specific acupuncture protocol for treating PCOS. This study will contribute to the clinical evidence regarding the effectiveness of electroacupuncture for women with PCOS.

## Trial status

The trial had not yet begun at the time of manuscript submission.

## Abbreviations

PCOS: polycystic ovary syndrome
EA: electroacupuncture
CC: clomiphene citrate
LH: luteinizing hormone
E2: estradiol
FSH: follicle-stimulating hormone
RCT: randomized controlled trial
CONSORT: Consolidated Standards of Reporting Trials
STRICTA: Standards for Reporting Interventions in Controlled Trials of Acupuncture
ESHRE: European Society of Human Reproduction and Embryology
ASRM: American Society for Reproductive Medicine
SAS: Statistical Analysis System
FAS: full analysis set
ITT: intention-to-treat
PPS: per-protocol analysis set
P25: percentile 25
P75: percentile 75
